# Refolding, Crystallization, and Crystal Structure Analysis of a Scavenger Receptor Cysteine-Rich Domain of Human Salivary Agglutinin Expressed in *Escherichia coli*

**DOI:** 10.1007/s10930-023-10173-x

**Published:** 2024-01-24

**Authors:** Changyu Zhang, Peng Lu, Sibo Wei, Chaoyue Hu, Mitsuko Miyoshi, Ken Okamoto, Hideaki Itoh, Suguru Okuda, Michio Suzuki, Hiroshi Kawakami, Koji Nagata

**Affiliations:** 1 Department of Applied Biological Chemistry, Graduate School of Agricultural and Life Sciences, The University of Tokyo, 1-1-1 Yayoi, Bunkyo-ku, Tokyo, 113-8657 Japan; 2 Department of Food Science and Nutrition, Faculty of Home Economics, Kyoritsu Women’s University, 2-2-1 Hitotsubashi, Chiyoda-ku, Tokyo, 101-8437 Japan; 3 Agricultural Bioinformatics Research Unit, Graduate School of Agricultural and Life Sciences, The University of Tokyo, 1-1-1 Yayoi, Bunkyo-ku, Tokyo, 113-8657 Japan; 4 Research Center for Food Safety, Graduate School of Agricultural and Life Sciences, The University of Tokyo, 1-1-1 Yayoi, Bunkyo-ku, Tokyo, 113-8657 Japan

**Keywords:** Crystal structure, Multiple disulfide bonds, Scavenger receptor, SRCRD, SHuffle T7, Refolding

## Abstract

Scavenger receptors are a protein superfamily that typically consists of one or more repeats of the scavenger receptor cysteine-rich structural domain (SRCRD), which is an ancient and highly conserved protein module. The expression and purification of eukaryotic proteins containing multiple disulfide bonds has always been challenging. The expression systems that are commonly used to express SRCRD proteins mainly consist of eukaryotic protein expression systems. Herein, we established a high-level expression strategy of a Type B SRCRD unit from human salivary agglutinin using the *Escherichia coli* expression system, followed by a refolding and purification process. The untagged recombinant SRCRD was expressed in *E. coli* using the pET-32a vector, which was followed by a refolding process using the GSH/GSSG redox system. The SRCRD expressed in *E. coli* SHuffle T7 showed better solubility after refolding than that expressed in *E. coli* BL21(DE3), suggesting the importance of the disulfide bond content prior to refolding. The quality of the refolded protein was finally assessed using crystallization and crystal structure analysis. As proteins refolded from inclusion bodies exhibit a high crystal quality and reproducibility, this method is considered a reliable strategy for SRCRD protein expression and purification. To further confirm the structural integrity of the refolded SRCRD protein, the purified protein was subjected to crystallization using sitting-drop vapor diffusion method. The obtained crystals of SRCRD diffracted X-rays to a resolution of 1.47 Å. The solved crystal structure appeared to be highly conserved, with four disulfide bonds appropriately formed. The surface charge distribution of homologous SRCRD proteins indicates that the negatively charged region at the surface is associated with their calcium-dependent ligand recognition. These results suggest that a high-quality SRCRD protein expressed by *E. coli* SHuffle T7 can be successfully folded and purified, providing new options for the expression of members of the scavenger receptor superfamily.

**Supplementary Information:**

The online version contains supplementary material available at 10.1007/s10930-023-10173-x.

## Introduction

The scavenger receptors are a protein superfamily that have been shown to be widely expressed in a variety of tissues [1] and to recognize diverse ligands [2]. The scavenger receptor family includes a range of secreted and membrane-associated proteins with widespread functions in the immune response [3], cell differentiation [4], apoptosis [5], and tumor suppression [6] that play an important role in host defense, such as sensing and cleaning various pathogens [1]. The scavenger receptors usually consist of one or more repeats of the scavenger receptor cysteine-rich domain (SRCRD) protein, which is an ancient and highly conserved protein module comprising about 110 residues [7]. SRCRD proteins have been classified into two types based on the number of cysteine residues [8]; Type A proteins are encoded by two exons and contain six cysteine residues, whereas Type B proteins typically contain eight cysteines and are encoded by one exon [9]. Although the number of cysteines differs, the relative pairing of SRCRDs from different sources is consistent [10].

The expression and purification of active and correctly folded SRCRD proteins are an important basis for crystallography studies of the scavenger receptors. To date, several crystal structures of the SRCRD from various scavenger receptor members have been determined, including Salivary scavenger and agglutinin (SALSA) [11], SCARA1 [12], SCARA5 [1], M2BP [13], MARCO [3], CD163 [14, 15], CD6 [16], CD5 [17], and hepsin [18]. The SRCRDs contain three or four pairs of disulfide bonds that are essential for maintaining the structural stability of the protein, which requires a protein expression system that has the ability to modify the disulfide bonds and fold the protein correctly. The expression systems commonly used in these studies to express SRCRD proteins were mainly an insect baculovirus expression vector system and a mammalian cell expression system, such as S2 cells [11, 14, 15] from *Drosophila*, High Five cells [1, 12] from *Trichoplusia ni*, and human HEK 293-EBNA embryonic kidney cells [15, 17]. Yeast expression systems have also been used for SRCRD expression, such as strain X-33 [15] and strain KM71 [18] from *Pichia pastoris* (Table 1). Although the eukaryotic expression systems offer the ability to express complex multi-disulfide-bonded proteins, such as SRCRD, these systems are slow, have a low yield, and are expensive [19].

**Table 1:** Eukaryotic expression systems of SRCRD crystal structures from scavenger receptor proteins

	Protein	Source	Sequence	Expression system	PDB
1	SRCR domain 1	SALSA	143	S2 cell (*Drosophila melanogaster*)	6SA4
2	SRCR domain 8	SALSA	137	S2 cell (*Drosophila melanogaster*)	6SA5
3	SRCR domain	Human SCARA1	113	High Five cell (*Trichoplusia ni*)	7DPX
4	SRCR domain	Human SCARA5	114	High Five cell (*Trichoplusia ni*)	7C00
5	SRCR domain	M2BP	119	HEK 293-EBNA cell (*Homo sapiens*)	1BY2
6	SRCR domain	Mouse MARCO	102	HEK 293-EBNA cell (*Homo sapiens*)	2OY3
7	SRCR domain 5	Porcine CD163	113	S2 cell (*Drosophila melanogaster*)	5JFB
8	CD163-like homolog SRCR8	CD163L1	104	S2 cell (*Drosophila melanogaster*)	6K0O
9	moCD163 SRCR5	Monkey CD163	107	X-33 cells (*Pichia pastoris*)	6K0L
10	Extracellular SRCR domains	Human CD6	371	CHO Lec 3.2.8.1 cell (*Cricetulus griseus*)	5A2E
11	CD5 domain III	CD5	101	HEK 293-EBNA cell (*Homo sapiens*)	2JA4
12	Extracellular domain	Human hepsin	114	KM71 (*Pichia pastoris*)	1P57
13	SRCR domain	Mouse SCARA1	117	High Five cell (*Trichoplusia ni*)	6J02
14	N-terminal SRCR domain	hLOXL2	457	HEK 293-EBNA cell (*Homo sapiens*)	5ZE3

Among the various expression systems available, *E. coli* has become the most widely used host for the production of recombinant proteins because of the advantages of rapid cell multiplication, high yield, and the relative ease of IPTG-induced expression [20]. However, most eukaryotic proteins usually suffer from insolubility and misfolding when expressed by the *E. coil* system, thus requiring the solubilization and refolding of the protein inclusion bodies [21]. Therefore, the expression of recombinant proteins containing multiple disulfide bonds in *E. coli* remains challenging. Some multi-disulfide-bonded proteins have been reported to be expressed in *E. coli* strains, resulting in the formation of inactive inclusion bodies that require renaturation to gain biological activity [22]. In the SHuffle strain, the periplasmic disulfide bond isomerase DsbC has been reported to shuffle the disulfide bonds within the mis-oxidized protein to produce its native folded state [23, 24], thus greatly enhancing the amount of correctly folded disulfide-bonded proteins [25].

SALSA, which is also as known as deleted in malignant brain tumors 1 (DMBT1) and salivary agglutinin (SAG), is a member of the scavenger receptor family [26] that was thought to be involved in the oral clearance of microorganisms because of its bacterial agglutination properties [27]. This protein contains 14 Type B SRCRDs separated by SRCR-interspersed domains (SIDs) [28]. Because of its ability to bind and agglutinate *Streptococcus mutans*, SALSA has been considered to play an important role in preventing dental caries [29]. In the present study, we developed an expression and purification method for the SRCRD from SALSA utilizing the Shuffle T7 *E. coli* expression system; moreover, we performed refolding to obtain diffraction-quality crystals during crystallization. The strategy developed in this study was considered to be reliable, as further confirmed by structural analysis, thus contributing to the structural and functional study of other scavenger receptor family proteins and cysteine-rich proteins.

## Materials and Methods

### Expression of the SRCRD Protein in *E. coil*

The DNA encoding SRCRD11 (1371–1489) of the deleted in malignant brain tumors 1 (DMBT1) protein was cloned into pET-32a in two forms: Trx-His6-TEV-SRCRD and SRCRD only for soluble and insoluble expression, respectively. In the first form, a thioredoxin (Trx) tag was added at the N-terminus for expression in the supernatant, followed by a polyhistidine (His6) tag for affinity purification. A Tobacco Etch Virus (TEV) protease cleavage site was added between the His6 tag and the SRCRD to remove the tags for further purification. The recombinant plasmids were transformed into *E. coli* SHuffle T7 (New England Biolabs) cells, which were then spread on an LB agar plate (100 µg/mL ampicillin). A single colony from the plate was picked up and precultured in 10 mL of LB medium (100 µg/mL ampicillin). The transformant was cultured overnight at 37℃ with shaking at 100 rpm and then transferred into 1 L of LB medium in a 5-L Erlenmeyer flask. The culture solution was incubated at 37 °C with shaking until the OD_600_ reached 0.6–0.8. Subsequently, the culture solution was rapidly cooled by placing it in ice water. Overnight expression was induced by 0.5 mM isopropyl-β-d-thiogalactopyranoside (IPTG) at 25 °C with shaking at 100 rpm. *E. coli* cells were harvested by centrifugation at 3500×*g* at 4 °C for 30 min. The harvested cells were collected in sampling bags, flash frozen in liquid nitrogen, and stored at − 80 °C.

### Refolding and Purification Strategy for SRCRD Expression in the Supernatant

The cell pellets were resuspended with lysis buffer (20 mM Tris–HCl (pH 8.0), 200 mM NaCl, and 10 mM imidazole) and sonicated to break the cells using a Digital Sonifier (Branson). The conditions of sonication were: Time = 7 min, Power = 60%, On = 1.0 s, Off = 1.0 s, max temperature = 7 °C. The lysate was centrifuged at 4 °C at 30,000 × g for 30 min, to pellet the cellular debris. The supernatant was then applied to nickel–nitrilotriacetic acid (Ni–NTA) agarose resin (Fujifilm), for purification according to the manufacturer’s protocol. The Ni–NTA resin was pre-equilibrated with lysis buffer. Next, the supernatant was mixed with resin for 30 min, to immobilize the target protein. After washing with washing buffer (20 mM Tris–HCl (pH 8.0), 200 mM NaCl, and 20 mM imidazole), the protein was eluted with elution buffer (20 mM Tris–HCl (pH 8.0), 200 mM NaCl, and 250 mM imidazole). The eluted protein was then buffer-exchanged with dialysis buffer (20 mM Tris–HCl (pH 8.0) and 200 mM NaCl) and digested with TEV protease at 4 °C overnight, to remove the Trx-His_6_-tag. A Resource RPC column (Cytiva) was used to separate the pure SRCRD protein from the digestion solution using a linear gradient of 1%–99% acetonitrile (1% trifluoroacetic acid). The target fractions were collected and freeze-dried. A refolding process was carried out at 4 °C in 50 mM Tris–HCl (pH 8.0), 0.2 M NaCl, 0.4 M l-Arg, 2.5 mM CaCl_2_, 2 mM/0.4 mM glutathione reduced form (GSH)/glutathione oxidized form (GSSG), and 10% glycerol. The refolded protein was then buffer-exchanged in 50 mM Tris–HCl (pH 8.0), 0.2 M NaCl, 2.5 mM CaCl_2_, and 10% glycerol for 24 h, twice.

### Refolding and Purification Strategy for SRCRD Expression in Inclusion Bodies

The cell pellets were suspended with lysis buffer (20 mM Tris–HCl (pH 8.0) and 200 mM NaCl) and sonicated to break the cells. The lysate was centrifuged at 4 °C at 30,000 × g for 30 min, to collect the inclusion bodies. The inclusion bodies were first washed twice with washing buffer (20 mM Tris–HCl (pH 8.0), 200 mM NaCl, 2 M urea, and 5 mM EDTA) and centrifuged at 4 °C at 8000 × g for 20 min to remove soluble impurities. The inclusion bodies were then solubilized in dissolution buffer (20 mM Tris–HCl (pH 8.0), 200 mM NaCl, and 8 M urea) with shaking at 4 °C overnight, and the supernatant was collected by centrifugation at 30,000 × g for 30 min at 4 °C. The refolding of SRCRD was performed through a dilution process with addition of the solubilized SRCRD sample to eightfold volume of refolding buffer (50 mM Tris–HCl (pH 8.0), 200 mM NaCl, 1 M urea, 0.5 M l-Arg, 2 mM GSH, 0.4 mM GSSG, and 2.5 mM CaCl_2_) in several batches. The final concentration of the protein was about 0.2 mg/mL. This refolding process was carried out over 1 week at 4 °C. After the refolding procedure, the sample was buffer-exchanged with dialysis buffer 1 (20 mM Tris–HCl (pH 8.0), 200 mM NaCl, and 50 mM Arg), followed by dialysis buffer 2 (20 mM Tris–HCl (pH 8.0)) at 4 °C. Subsequently, the refolded SRCRD was filtered and further loaded onto a Mono Q column (GE Healthcare) to remove protein impurities using an ÄKTA purifier (GE Healthcare) as the final purification. Elution was performed with a linear gradient of 0–1.0 M NaCl in 20 mM Tris–HCl (pH 8.0) buffer.

### MALDI-TOF MS

Sinapinic acid, which was used as the matrix solution, was dissolved in the mixture solution of TA30 (acetonitrile and 0.1% TFA mixed at a ratio of 30:70). Proteins were diluted with 0.1% TFA to a concentration of about 10 μM and mixed with matrix at a ratio of 1:1. The mixed solution was spotted onto a metal plate and dried by air before being loaded onto an AutoFlex Speed MALDI-ToF instrument (Bruker). The analytical mode was set to LP m/z HighMasses. Bovine serum albumin was used as the calibration reagent. The laser power was 70%, and spotting was performed about 20 times for each sample. Spectra were analyzed using the flexAnalysis Software (Bruker).

### Crystallization

The purified SRCRD protein was concentrated to 12 mg/mL using a 5000 MWCO VIVASPIN (Sartorius) ultrafiltration device, followed by screening of the crystallization conditions using Wizard I & II (Rigaku Reagents, Inc.), Crystal Screen HT (Hampton Research), and PEG/Ion HT (Hampton Research) kits. The sitting-drop vapor diffusion method was adopted at 20 °C in 96-well VIOLAMO Protein Crystallization Plates (As One); 0.5 µL of the protein solution and 0.5 µL of the reservoir solution were mixed in one drop, for crystal growth. SRCRD expressed in the supernatant formed needle-shaped crystals under the following crystallization conditions: 0.2 M lithium citrate tribasic tetrahydrate, 25% PEG 3350, pH 7.0. SRCRD expressed in inclusion bodies formed rhombic crystals under the following crystallization conditions: 0.2 M ammonium iodide, 20% PEG 3350, pH 6.2. These crystals were used for data collection.

### X-ray Diffraction Data Collection and Processing

The crystals were picked up with a Mounted CryoLoop (Hampton Research) and cryoprotected in a reservoir solution containing 20% glycerol. Subsequently, the crystals were flash frozen in liquid nitrogen and transferred to unipuck (crystal positioning system). X-ray diffraction data of the SRCRD crystals were collected on beamline AR-NE3A at Photon Factory (Ibaraki, Japan) at a wavelength of 1.000 Å. The diffraction data were indexed and integrated using the XDS program [30] and then scaled using XSCALE [30]. The crystal structure of the SRCRD protein was solved by molecular replacement with MOLREP [31, 32] from the CCP4 suite, using the structure of GP340 SRCRD 8 (PDB 6SA5; sequence identity: 96.33%) as an input model. The building of a model of the crystal structure of SRCRD was carried out by CCP4i and Coot [33, 34]. The structure was further refined using Refmac5 [35] and Phenix refine [36].

### Size Exclution Chromatography

The protein sample was concentrated to 1 mL and loaded onto a Superdex 200 10/300 GL column (GE Healthcare) with gel filtration buffer (20 mM Tris–HCl (pH 8.0), and 200 mM NaCl) using an ÄKTA purifier (GE Healthcare).

## Results

### Refolding and Purification of SRCRD Expressed in the Supernatant of an *E. coil* Lysate

**Fig. 1:**
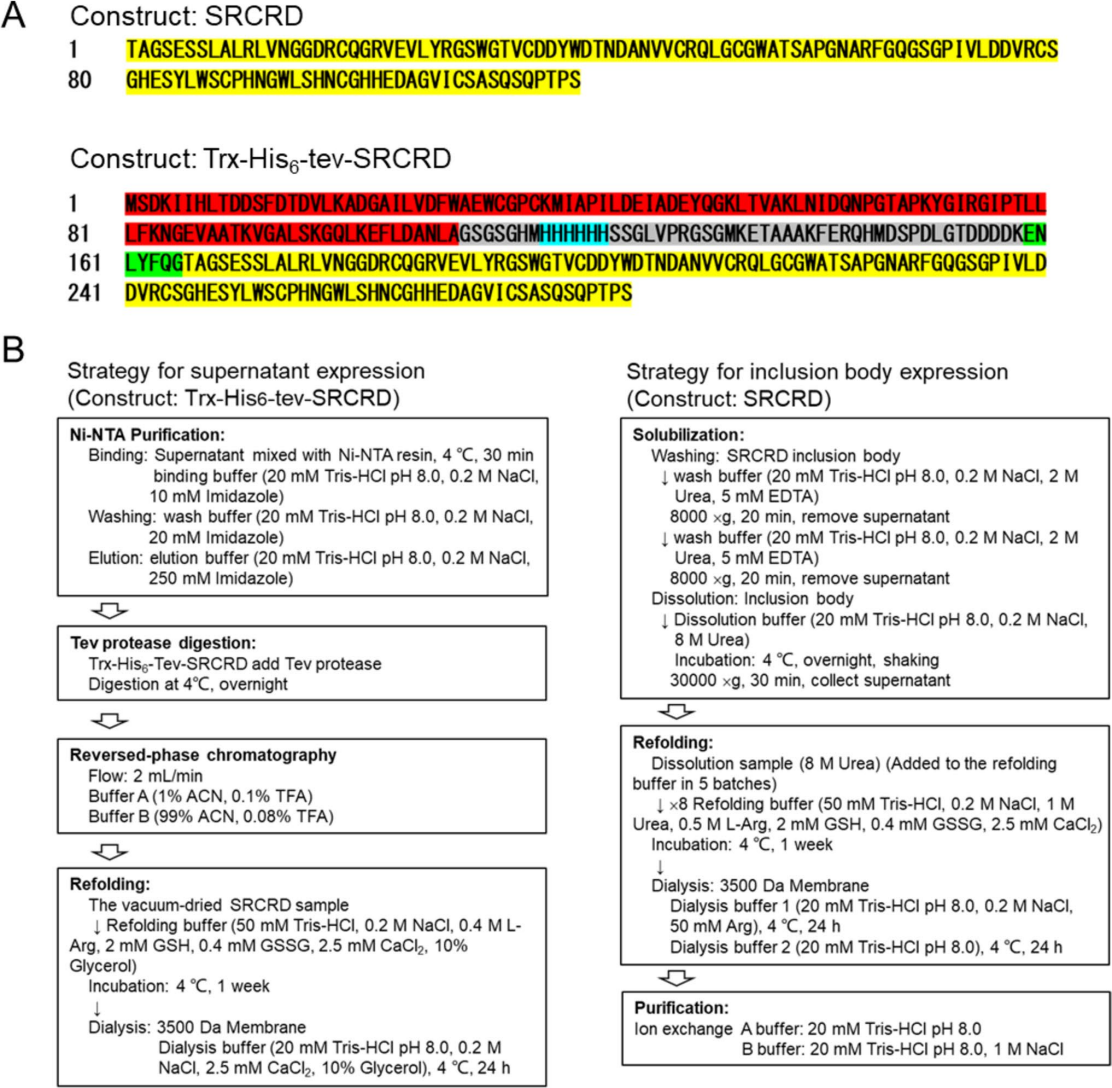
Strategies used for the expression, purification, and refolding process of SRCRD. **A** Amino acid sequence of the constructs used for SRCRD expression. The SRCRD construct was used for inclusion body expression. The Trx-His_6_-TEV-SRCRD construct was used for supernatant expression. The red box indicates the thioredoxin tag, the blue box indicates the His affinity tag, the green box indicates the TEV protease cleavage site, and the yellow box indicates the SRCRD. **B** Protocols used for the purification and refolding process of SRCRD expressed in supernatant and inclusion bodies

**Fig. 2:**
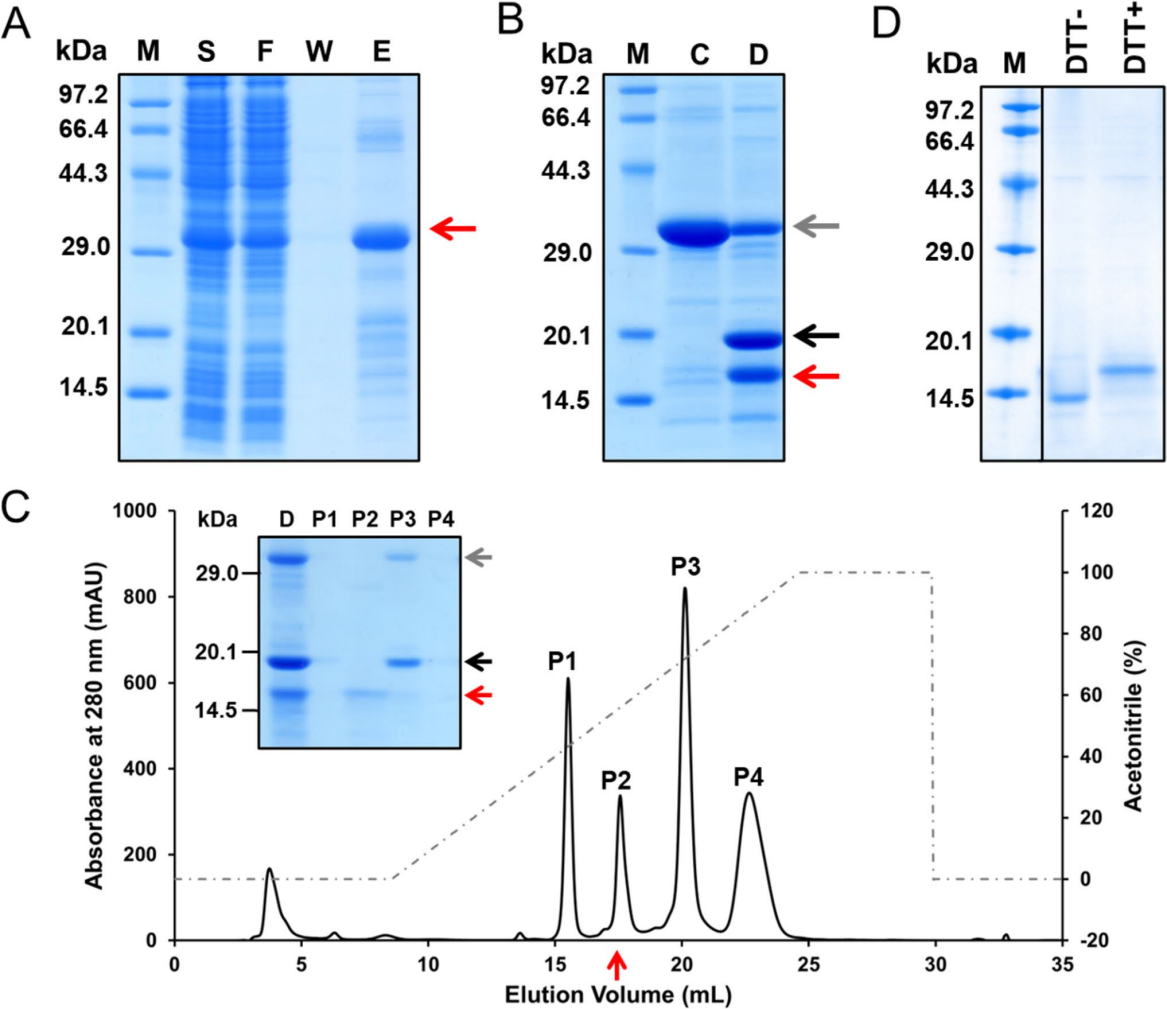
Refolding and purification of SRCRD expressed in the supernatant of an *E. coil* lysate. **A** SDS-PAGE for Trx-His_6_-TEV-SRCRD expression confirmation and purification by Ni–NTA chromatography. “S” represents the soluble fraction of the cell lysate. “F” represents the sample after flowing through the Ni–NTA resin. “W” represents the sample after rinsing with washing buffer. “E” is the fraction eluted by the elution buffer. **B** SDS-PAGE of TEV protease digestion for the production of SRCRD. “C” represents the Trx-His_6_-TEV-SRCRD sample before the addition of TEV protease. “D” represents the sample after digestion by TEV protease at 4 °C overnight. **C** SRCRD from the TEV protease digestion product was purified by reverse-phase chromatography. SDS-PAGE was used to visualize the proteins in each peak separated by RPC. **D** SDS-PAGE of purified SRCRD after refolding was treated with non-reducing sample buffer (no DTT) and reducing sample buffer (0.2 M DTT), respectively. Proteins containing disulfide bonds exhibit a loose state because of the breakage of the disulfide bonds by DTT; thus, they move more slowly through SDS-PAGE than do proteins with intact structures. The gray arrow indicates the band of Trx-His_6_-TEV-SRCRD, the black arrow indicates the band of Trx-His_6_ tag, and the red arrow indicates the band of SRCRD

To express the SRCRD protein in the soluble fraction, we first attempted a strategy to improve the solubility of the expressed proteins by fusing a thioredoxin (Trx) tag [37] to the N-terminal end of the construct using pET-32a (Fig. 1A). A histidine tag for affinity purification followed the Trx-tag. Moreover, a TEV protease cleavage site replaced the enterokinase cleavage site between the histidine tag and the SRCRD sequence to allow subsequent digestion with TEV protease to obtain the SRCRD protein. The target protein (Trx-His_6_-TEV-SRCRD) was expressed in the soluble fraction of the *E. coli* SHuffle T7 lysate and observed as a band of about 30.5 kDa on SDS–PAGE (Fig. 2A).

The soluble fraction containing Trx-His_6_-TEV-SRCRD from the *E. coil* lysate was initially separated by immobilized Ni–NTA affinity chromatography. Trx-His_6_-TEV-SRCRD was successfully trapped on the Ni–NTA resin via the binding affinity of the His_6_ tag to Ni^2+^ ions. Most of contaminant proteins were removed by the washing buffer, and the target protein was eluted using a solution containing 250 mM imidazole. The identity of Trx-His_6_-TEV-SRCRD protein was further confirmed by MALDI-TOF–MS (Fig. S1). TEV protease digestion was then performed to cut the protein at the cleavage site and separate the Trx-His_6_ tag from the SRCRD protein. The SRCRD protein without the tags was confirmed by SDS-PAGE (Fig. 2B). However, this digestion was inadequate, and a large amount of protein remained from which the excess tags could not be removed, which reduced the yield of SRCRD. This incomplete digestion may be attributed to the formation of protein aggregates, resulting in the inability of TEV protease to effectively act at the cleavage site. Non-reducing SDS–PAGE revealed the formation of aggregates, and the administration of DTT, a reducing agent that is often used in protein purification, eliminated this state (Fig. S2). These results suggested that intermolecular disulfide bonds were likely to be the contributing factor in the formation of the aggregates. Although the protein was solubilized with the help of Trx tags, the disulfide bonds within the recombinant protein molecule remained incorrectly folded.

Reverse-phase chromatography was selected for the purification of SRCRD from the digestion mixture, and the bands were confirmed by SDS–PAGE (Fig. 2C). As the content of the organic phase increased, the SRCRD appeared in peak 2 at approximately 55% acetonitrile (ACN). The Trx-His_6_ tag and the uncleaved protein were separated into peak 3. The peak 2 fraction samples were then freeze-dried, to remove the organic solvents.

Freeze-dried samples from RPC were prone to precipitation when redissolved in buffer, which may be attributed to incorrect folding of the disulfide bonds. Loading of the RPC-purified SRCRD sample into the SEC revealed the presence of many aggregates in the sample (Fig. S3). An incomplete structure with free cysteine residues may have led to this result. To improve its quality, the SRCRD sample needs to be refolded to break the intermolecular disulfide bonds of the protein and form correct intramolecular disulfide bonds. Because DTT is too reductive and may break the otherwise correctly formed disulfide bonds in proteins, glutathione was chosen as the redox system for the refolding process because of its stable and reversible effect. In this study, the disulfide bonds of SRCRD were refolded using a refolding buffer containing GSH/GSSG as a redox system, l-arginine, calcium ion, and glycerol, simultaneously. After long period of refolding, the number of aggregates in the sample was decreased (Fig. S3). The purified SRCRD after refolding was checked by non-reducing (no DTT) and reducing (0.2 M DTT) SDS–PAGE (Fig. 2D). Proteins containing disulfide bonds may exhibit a loose state when the disulfide bonds are broken by DTT; thus, they move more slowly in SDS–PAGE than do the proteins with intact structures. The addition of DTT caused a change in the position of the SRCRD protein on SDS–PAGE, which demonstrated that disulfide bonds had been formed within the refolded SRCRD.

### Refolding and Purification of SRCRD Expressed from the Inclusion Bodies of an *E. coli* Lysate

**Fig. 3:**
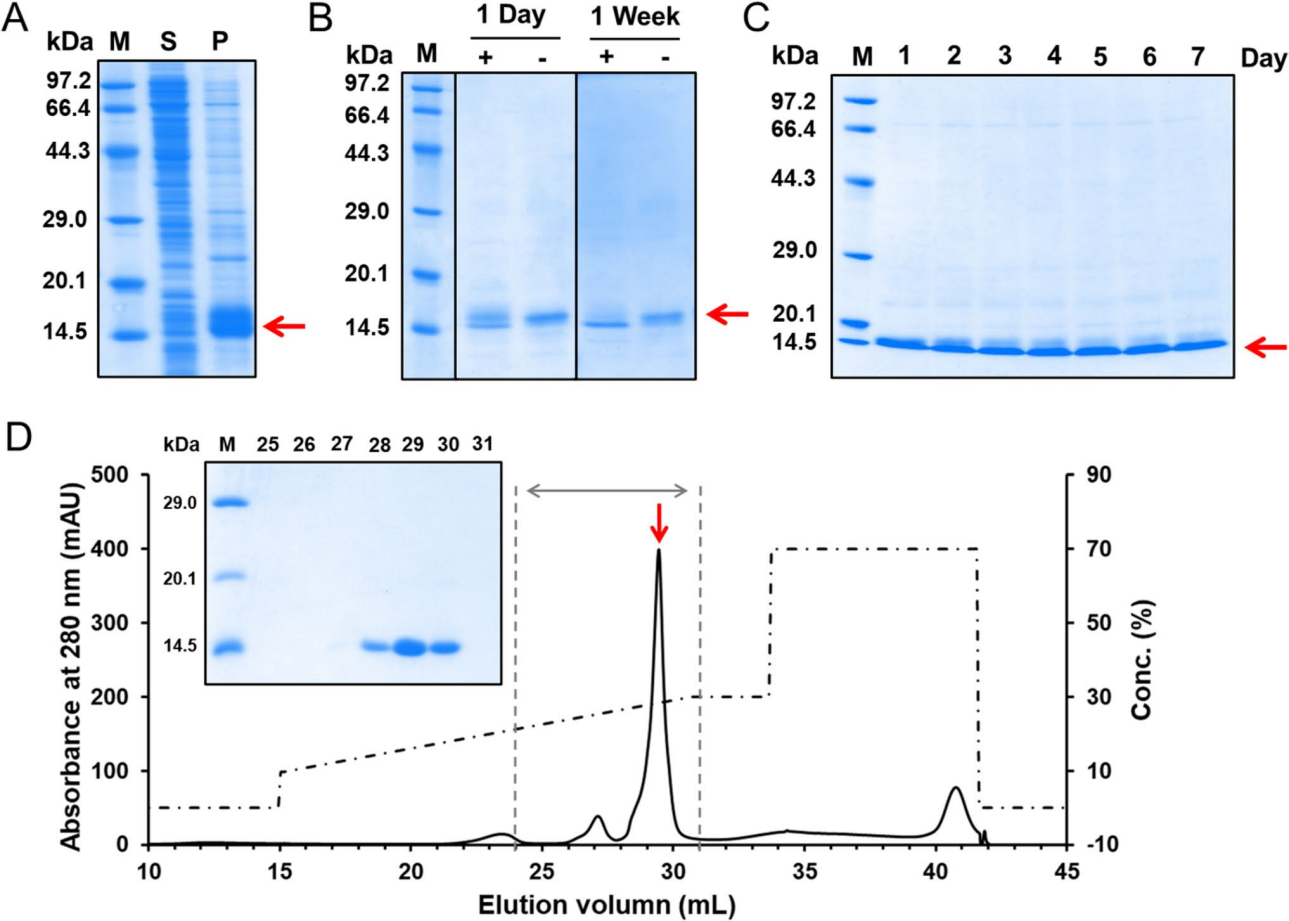
Refolding and purification of SRCRD expressed in inclusion bodies. **A** The band of SRCRD was confirmed in the precipitate by SDS–PAGE. “S” represents the supernatant fraction of the cell lysate. “P” represents the precipitate fraction of the cell lysate. **B** SRCRD was treated by short (1 day) and long (1 week) refolding with (+) and without (−) GSH/GSSG, respectively. **C** The GSH/GSSG redox system gradually reduced the unfolded protein over the span of 1 week. The upper bands are unfolded proteins, whereas the lower bands are refolded proteins. **D** Refolded SRCRD was purified by anion exchange chromatography using a Mono Q column, and SDS–PAGE shows the eluted fractions (marked by gray dotted lines) corresponding to the highest peak. The SRCRD protein is indicated by a red arrow. SDS-PAGE was checked with non-reducing sample buffer

Even if the protein is expressed as inclusion bodies, some methods (such as solubilization and refolding) can be chosen to restore the solubility and natural structure of the protein. To apply this strategy to the expression of SRCRD, a construct containing only SRCRD without tags was used for SRCRD expression (Fig. 1A). In the absence of a Trx tag to enhance the solubility of the expressed protein, the SRCRD proteins were expressed as inclusion bodies in the precipitate of the *E. coli* lysate after centrifugation; furthermore, the target protein in the inclusion bodies was observed as a band of about 12.5 kDa on SDS–PAGE (Fig. 3A).

The precipitate containing SRCRD inclusion bodies was first washed twice with washing buffer, then centrifuged to remove soluble impurities. The inclusion bodies of SRCRD were retained and then solubilized in dissolution buffer containing 8 M urea with shaking at 4 °C overnight, followed by the removal of the insoluble matter by centrifugation. The solubilized SRCRD sample was added to refolding buffer contain GSH/GSSG or no GSH/GSSG in a stepwise manner and with sufficient stirring to begin the refolding process. This refolding process then lasted for 1 week at 4 °C, and the refolded protein samples were checked by SDS–PAGE (Fig. 3B). Two SRCRD bands appeared on the first day of refolding with GSH/GSSG, indicating that some of the SRCRD had been refolded; however, a fraction of the SRCRD remained in a loose state because the intramolecular disulfide bonds were not all formed. The SRCRD obtained after refolding without GSH/GSSG had only one upper band, indicating that the protein was not efficiently refolded. After refolding for 1 week, with the help of the GSH/GSSG, most of the SRCRD transformed into a lower band. In contrast, in the refolding without GSH/GSSG, most of the SRCRD remained as the same upper band. Samples were collected daily during the refolding process and checked by SDS–PAGE (Fig. 3C). The upper unfolded bands gradually disappeared over time (days), eventually leaving only the lower bands, representing the refolded protein. The sample was finally dialyzed to remove all chaotropic agents and redox reagents and end the refolding process. The refolded protein was loaded onto the SEC and a large peak representing the monomer was eluted (Fig. S4), indicating that most of the protein was retained as a monomer after refolding. Together, these results provide important insights into the importance of the GSH/GSSH redox system for the refolding of multi-disulfide-bonded proteins. Another *E. coli* strain, BL21(DE3), was used for SRCRD expression; however, it failed to yield soluble protein after refolding attempts. Precipitation of proteins occurred when refolding reagents were removed using dialysis (Fig. S5). Thus, SHuffle T7 is more suitable for SRCRD expression than the commonly used BL21(DE3) strain.

For crystallization experiments, further ion exchange chromatography was used for the purification of the SRCRD protein. After the refolding process, the SRCRD protein sample was buffer-exchanged by dialysis at 4 °C for 48 h, to remove all the salt present in the sample. Subsequently, the refolded SRCRD was filtered and further loaded onto a Mono Q column, to remove protein impurities as the final purification (Fig. 3D). The purity of the target protein was confirmed by SDS–PAGE, and the identity of the protein was further confirmed by MALDI-TOF–MS (Fig. S1).

### Crystallization and X-ray Diffraction of SRCRD

The purity and stability of a protein are essential prerequisites for its successful crystallization; thus, crystallization can be used to evaluate the SRCRD purification and refolding strategies propose here. To confirm further the structural integrity of the SRCRD protein and whether the disulfide bonds in the protein were formed correctly after refolding, the crystallization experiments of SRCRD were performed using the sitting-drop vapor diffusion method. The purified SRCRD was concentrated to 12 mg/ml on an ultrafiltration device, and several commercial crystallization screening kits were used in the crystallization screening experiment.

**Fig. 4:**
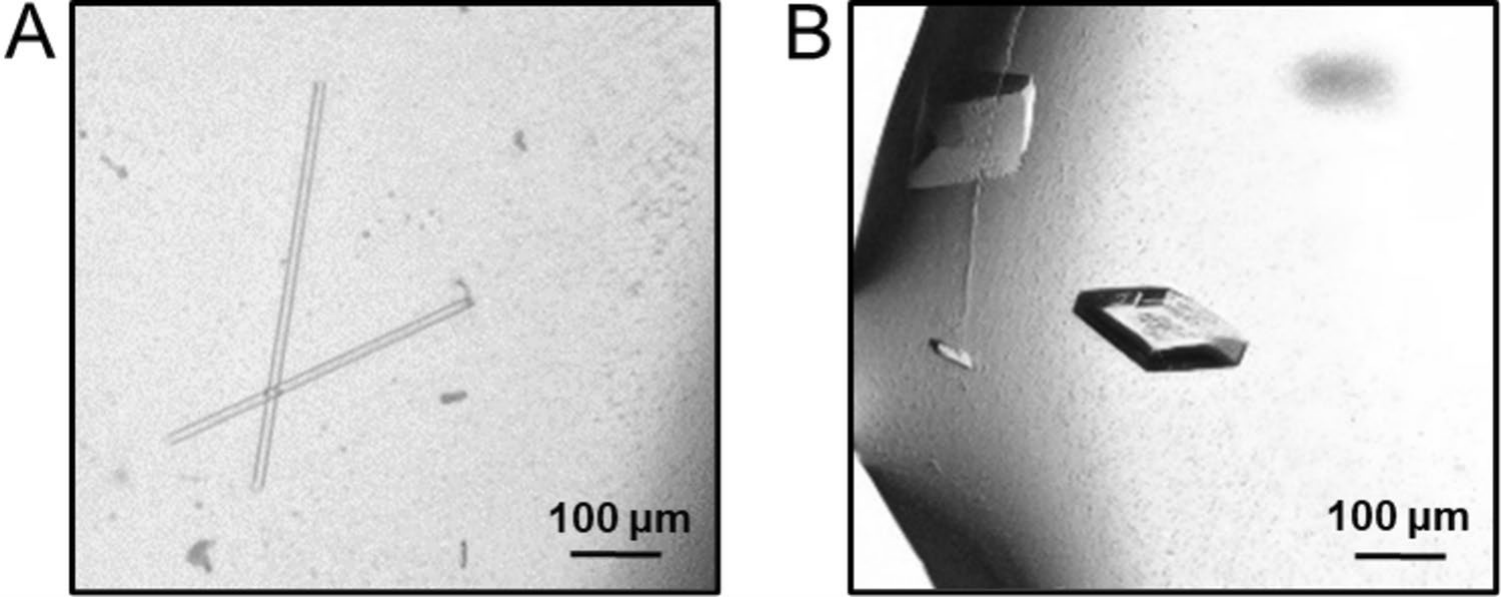
Two types of crystals formed by the SRCRD protein. **A** Noodle-shaped crystals were formed by SRCRD expressed in the supernatant. The crystallization condition was as follows: 0.2 M lithium citrate tribasic tetrahydrate, 25% w/v polyethylene glycol 3,350, pH 7.0. **B** Rhombic crystals of SRCRD were formed by SRCRD expressed in inclusion bodies. The crystallization condition was as follows: 0.2 M ammonium iodide, 20% PEG3350, pH 6.2

More than 1 month after the SRCRD protein was purified and refolded from supernatant expression, a few protein crystals were finally detected in the reservoir solution containing lithium citrate tribasic tetrahydrate and polyethylene glycol 3,350. Noodle-shaped crystals formed in the condition with 0.2 M lithium citrate tribasic tetrahydrate, 25%–30% w/v polyethylene glycol 3,350, pH 6.0–7.0 (Fig. 4). However, the crystallization of SRCRD prepared using this purification and refolding strategy was not very reproducible, and crystals did not always grow. Therefore, this purification and refolding strategy for SRCRD expressed in supernatants is not adequately effective.

In the case of the SRCRD protein purified and refolded from inclusion body expression, the protein yields were enhanced, and large quantities of crystals could be obtained within a short period (2–3 days) in crystallization experiments. The growth of many crystals could be observed under several conditions using the PEG/ion kit. Most of these crystals were rectangular or rhombic in shape (Fig. 4). The crystallization experiments were repeated, and crystals were obtained every time using the PEG/ion kit (the conditions under which crystals often grew are shown in Fig. S6. Therefore, this strategy of purification and refolding of SRCRD proteins from inclusion bodies was considered to be reliable.

Whether the four pairs of disulfide bonds in SRCRD were correctly formed was further confirmed by X-ray diffraction experiments. The X-ray diffraction data of two types of SRCRD crystals (noodle-shaped and rhombic crystals) were collected at beamline AR-NE3A of Photon Factory at a wavelength of 1.000 Å. The diffraction data of a crystal with a high resolution were used for further analysis. The data collection statistics are listed in Table 2.

**Table 2:** X-ray diffraction data collection statistics

Data collection	Needle-shaped (PDB ID: 8WZS)	Rhombus (PDB ID: 8J8D)
Detector	AR-NE3A	AR-NE3A
Wavelength (Å)	1.000	1.000
Space group	*P* 3_2_ 2 1	*P* 2_1_ 2_1_ 2
Resolution (Å)	41.18–2.54 (7.51–2.54)	40.90–1.47 (4.39–1.47)
*Unit cell parameters*		
*a*, *b*, *c* (Å)	71.08, 71.08, 55.36	81.80, 31.70, 35.22
*α*, *β*, *γ* (º)	90, 90, 120	90, 90, 90
Total reflections	52,657 (8237)	74,507 (10,525)
Unique reflections	10,304 (1637)	22,788 (3604)
Completeness (%)	99.73 (99.51)	98.7 (96.8)
Redundancy	5.11 (5.03)	2.94 (2.64)
*R*_merge_ (%)	8.2 (101.2)	3.2 (6.5)
*R*_pim_ (%)	3.7 (5.1)	2.1 (2.7)
*CC 1/2* (%)	99.8 (63.4)	99.9 (98.6)
< *I* / σ (*I*) >	13.12 (1.49)	24.65 (8.11)

The highest resolution of the noodle-shaped crystal obtained here was determined to be 2.54 Å, and one asymmetric unit contained one molecule. The space group of the noodle-shaped crystal was determined to be *P* 3_2_ 2 1, with unit cell parameters of *a* = 71.08 Å, *b* = 71.08 Å, and *c* = 55.36 Å. The highest resolution of the rhombic crystal obtained was determined to be 1.47 Å, and one asymmetric unit contained one molecule. The space group of the rhombic crystal was determined to be *P* 2_1_ 2_1_ 2, with unit cell parameters of *a* = 81.80 Å, *b* = 31.70 Å, and *c* = 35.22 Å.

A structural analysis of the rhombic crystal was performed because of its higher resolution, and the structure refinement statistics are listed in Table 3. The initial phase of the SRCRD structure was established by the molecular replacement method using the PDB ID of the 6SA5 crystal structure as the search model. The initial model was determined, which was further refined using Refmac5, Coot, and Phenix. After refinement, the *R*_work_ and *R*_free_ of the final structure of SRCRD were refined to 15.8% and 17.6%, respectively. The Ramachandran plot indicated that the percentage of residues of SRCRD in preferred regions, allowed regions, and outliers was 96.32%, 3.68%, and 0.00%, respectively.

**Table 3:** Structure refinement parameters

Refinement parameters	Needle-shaped (PDB ID: 8WZS)	Rhombus (PDB ID: 8J8D)
*R*_work_/*R*_free_ (%)	17.59/24.29	15.8/17.6
*No. of atoms*		
Protein atoms	807	813
Water	83	154
Ions (Cl^−^)	0	2
Wilson B-factors (Å^2^)	63.2	11.3
*R.m.s. deviations*		
Bond length (Å)	0.0080	0.0107
Bond angles (º)	1.018	1.7136
*Ramachandran plot*		
Favored (%)	92.38	96.32
Allowed (%)	7.62	3.68
Outliers (%)	0.00	0.00

### Crystal Structure of the SRCRDs from SALSA

**Fig. 5:**
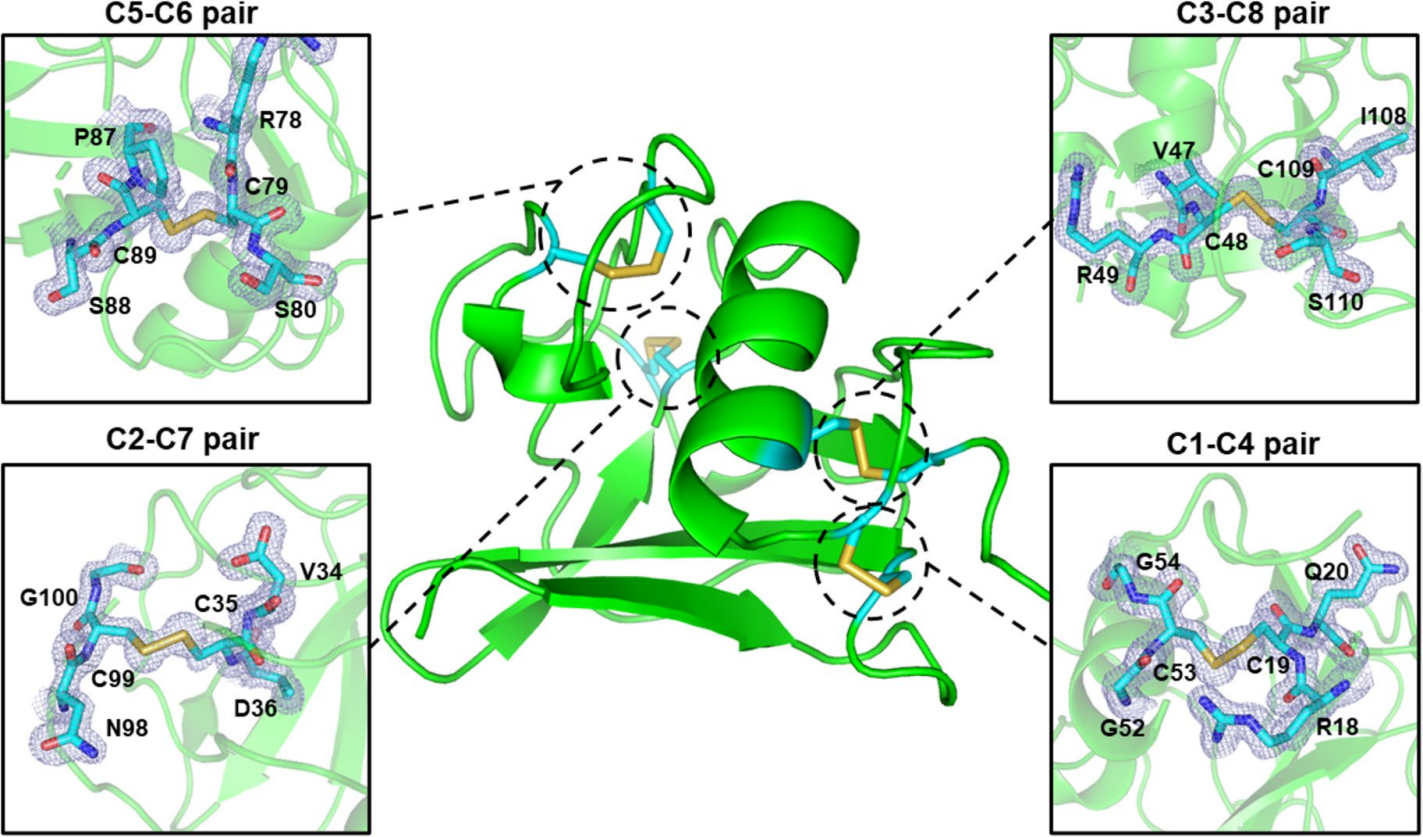
Confirmation of the presence of four disulfide bonds in the refolded SRCRD structure. The figure on the center is a schematic representation of the overall structure of SRCRD, whereas the four close-up views of the disulfide bond sites in the SRCRD structures are presented in stick annotation. The 2*Fo–Fc* map of the cystine residues and adjacent residues is contoured at the 1.0 σ level. The residues are shown in stick annotation in cyan. The disulfide bonds are shown in stick annotation in yellow. The structure images were created using Pymol

All known SRCRDs of scavenger receptor superfamily members share a very high degree of identity at the sequence and structural levels, which is even more evident in the SRCRDs from SALSA. The sequence of SRCRD11 solved in this research exhibited high similarity to another two SRCRDs with reported structures: SRCRD1 (PDBid: 6SA4, 90.00% identity) and SRCRD8 (PDBid: 6SA5, 96.33% identity); moreover, the fold was also very highly conversed. The crystal structure revealed a globular SRCR-fold containing four disulfide bridges with typical characteristics of Type B members (Fig. 5). The fold contained one α-helix at the center of the sequence and several β-strands at both the N and C termini. These β-strands linked the N and C termini together, to maintain a compact fold structure. The SRCR11 model spanned 96 residues and lacked the N-terminal “TAGSES” and C-terminal “QSQPTPS” sequences because of the high mobility of these loop tails. The refined electron density map was perfectly matched to the disulfide bonds, indicating that the eight cysteines formed the correct four pairs of disulfide bonds in SHuffle T7-expressed SRCR11, which ensured the success of the refolding process. As a Type B scavenger receptor protein, the SRCRD of SALSA has one additional disulfide bond pair compared with Type A domains, i.e., the C1–C4 pair, which corresponded to Cys19–Cys53 in the SRCR11 structure. The relative numbering of the remaining pairs was structurally conserved, with Cys35–Cys99 corresponding to the C2–C7 pair, Cys79–Cys89 corresponding to the C3–C8 pair, and Cys35–Cys99 corresponding to the C5–C6 pair. The C1–C4 and C3–C8 pairs are involved in linking the N- and C-terminal peptide chains and the α-helix, thus maintaining structural closure and compactness. The remaining two pairs of disulfide bonds are responsible for stabilizing the loop-rich flexible region.

### Comparison of Type A and B SRCRD

**Fig. 6:**
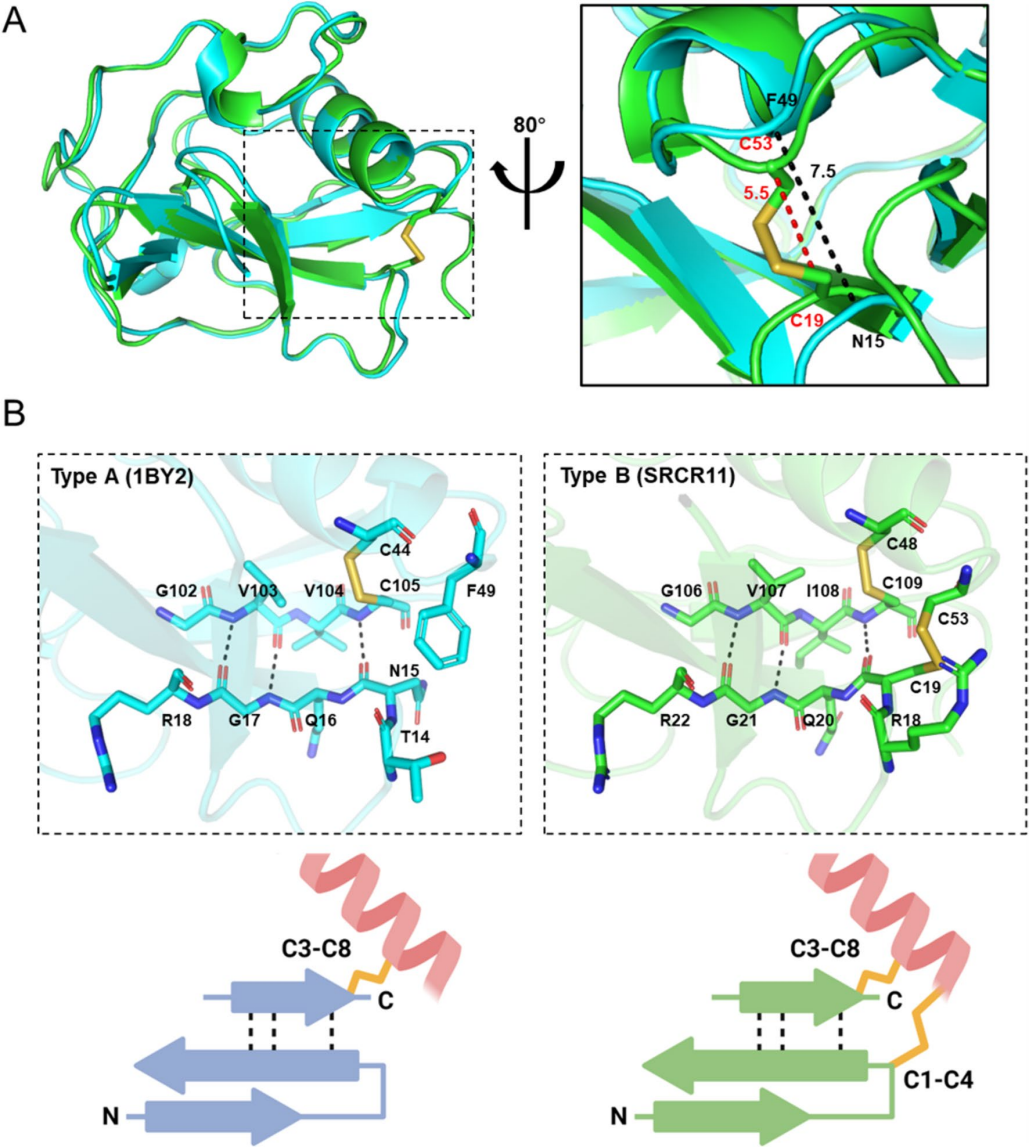
Difference in the C1–C4 pair disulfide bond between Type A (M2BP, cyan) and Type B (SRCR11, green) SRCRD. **A** The C1–C4 pair is present exclusively in Type B SRCRD. The regions involved in the formation of the N and C-terminal β-sheet are indicated by the dashed boxes. The map on the right is a close-up view of C1–C4 pair sites overlapped by two structures. The distance of Cys19 and Cys53 from the SRCR11 structure is indicated by the red dotted line. The distance of Asn15 and Phe49 from 1BY2 is indicated by the black dotted line. **B** A close-up view of the C1–C4 pair and adjacent β-strands. The residues are shown in stick annotation. The hydrogen bonds of the β-sheet are indicated by the black dotted line. The disulfide bonds are shown in stick annotation in yellow. The structure images were created using Pymol. The schematic diagrams of the structures are shown at the bottom of the figure

The disulfide bonds inside the molecules of SRCRDs play an important role in maintaining the structural stability and compactness of these proteins. The C1–C4 disulfide bond pair distinguishes Type A from Type B SRCRDs. This C1–C4 pair is located near the surface of the protein, which may be a factor in the production of inclusion bodies caused by intermolecular disulfide bonds during the expression of Type B SRCRD by SHuffle T7 cells. To understand the role of the C1–C4 pair in the structure of the protein, we compared the structure of Type B SRCR11 to that of Type A (the SRCRD from M2BP was used as a Type A template) regarding the C1–C4 pair and the adjacent β-sheet (Fig. 6A). The C1–C4 pair connects the second β-strand from the N terminus to the end of the α-helix. The disulfide bond formed by the Cys19–Cys53 pair in the structure of SRCR11 renders the structure more compact. In contrast, Asn15 and Phe49 of Type A SRCRD at the same amino acid sequence site are unable to form a bond and render the structure looser. In a close-up view of the C1–C4 pair and adjacent β-strands (Fig. 6B), the second β-strand located at the N-terminal of Type A formed three hydrogen bonds with the β-strand located at the C-terminal. Moreover, Cys105 at the C-terminal formed a C3–C8 pair disulfide bond with Cys44 on the α-helix, thus causing the two ends to establish a compact closure. In the Type B structure, in addition to the hydrogen bonds between the β-strands, Cys19 next to the second β-strand at the N terminus formed a C1–C4 pair with Cys53 at the end of the α-helix, which provides stronger support for the compactness of the terminal structure.

## Discussion

Tandem repeats of SRCRDs, similar to immunoglobulin (IG) and epidermal growth factor-like (EG) domains, are common among the membrane-bound members of the superfamily [13, 38, 39]. SRCRDs have been identified in numerous cell surface and secreted proteins, most of which are associated with the immune system, mediating protein–protein interactions and ligand binding [8]. In previous studies on SRCRD, eukaryotic expression systems were usually used to express these multi-disulfide-bonded proteins (Table 1) to ensure that the small and compact structure can be folded correctly. However, high costs, harsh culture conditions, susceptibility to contamination, and low protein yields represented obstacles to the use of eukaryotic cells in this context [40, 41], especially in structural studies [19, 42]. In contrast, bacterial cells have the advantage of being cost effective and easy to handle and modify. However, they are limited in the expression of large proteins and lack eukaryotic post-translational modifications [19].

To meet the requirements for a high protein yield and quality of various experiments, such as crystallization and NMR analysis, here, we aimed to identify an efficient and low-cost method to produce SRCRD proteins. An SRCRD unit (1371–1489) from human SALSA, a member of the Type B scavenger receptor subfamily, was targeted in this study. This SRCRD protein contains four pairs of disulfide bonds, which is a challenge for the *E. coli* expression system. *E. coli* SHuffle T7 cells, which are particularly adapted to facilitate disulfide bond formation, were used as the expression system for SRCRD. The SHuffle T7 strain is an *E. coli* K12 strain engineered to form proteins containing disulfide bonds in the cytoplasm and suitable for T7 promoter driven protein expression. In addition to mutations in *trxB* and *gor*, *DsbC* in the cytoplasm facilitates the formation of accurate disulfide bonds of the expressed proteins [23]. To express the SRCRD protein, its cDNA was inserted into the pET32a expression vector and expressed by SHuffle T7. One Type A SRCRD, the third SRCRD of murine neurotrypsin, has been reported to be expressed in the soluble fraction from SHuffle T7 cells [9]. However, because the Type B SRCRDs contain four pairs of disulfide bonds, the presence of eight cysteines can contribute significantly to the incorrect formation of disulfide bonds in the reducing environment of the *E. coli* cytoplasm [19]. It is also difficult for the SHuffle strain to support the solubility of the misformed protein. In our case, the SRCRD from human SALSA could not be expressed in the soluble fraction of the SHuffle T7 lysate and remained in the precipitate. The extra pair of disulfide bonds over Type B domains is likely to have contributed to this result.

Our initial attempt to produce the protein included fusing a Trx tag at the N terminus of the sequence, to increase the solubility of the recombinant protein, followed by digestion with TEV protease, to obtain purified SRCRD. However, the proteins obtained contained aggregates, thus requiring further refolding. To investigate the cause of protein agglutination, DTT, which is a reducing agent that is often used in protein purification, was added to the digested samples, followed by SDS–PAGE using a non-reducing sample buffer (Fig. S1). After the DTT treatment, the bands of the aggregate were disassembled, and the bands of SRCRD appeared at the right position. DTT is often used for the reduction of disulfide bonds in proteins and can be employed to block the formation of intermolecular disulfide bonds between cysteines in proteins. These results indicated that protein aggregation was the result of the incorrect disulfide bonding of cysteines between protein molecules and that the addition of reducing agents is effective in reducing the formation of aggregates. In cysteine-rich proteins, misconnection of disulfide bonds may lead to the formation of protein multimers. During refolding, the oxidation of disulfide bonds can be triggered by the thiol group of glutathione [43]. It has been reported that chitinase, which is a cysteine-rich protein from *E. coli* inclusion bodies, can be solubilized and correctly folded into active proteins in a glutathione redox system [44]. Therefore, further refolding was performed using the GSH/GSSG redox system, for the formation of appropriate disulfide bonds by the cysteines of SRCRD. However, the final yield of the purified protein was not high, probably because of the tedious steps and inadequate digestion process. Although crystals were finally obtained, the crystallization could not be stably repeated.

Subsequently, we attempted to express the protein in inclusion bodies, followed by solubilization with a high concentration of urea and arginine, as chaotropic agents. After removing all insoluble impurities, refolding was performed using the GSH/GSSG redox system. The final enrichment and purification of the protein were finished by ion exchange chromatography. This strategy ensured the quality of the recombinant protein while improving its yield. High resolution crystals were formed under a variety of crystallization conditions from a commercial PEG/ion kit within a few days. Because the constructs used in this method do not carry added tags, including the His tag, the minimization of the flexible region of the protein facilitates the formation of crystals. Additionally, we tried the construct His_6_-SRCRD by adding a histine tag at the N-terminal. High yield and purity of protein were obtained in case of SRCRD construct without any tags; however, no crystal growth was observed (Table 4). This may be due to the excess of terminal loop structure that was preventing protein crystal formation. Moreover, the BL21(DE3) strain was tested for its ability to express SRCRD; however, it failed to yield soluble protein from refolding. This may be due to the absence of the isomerase that promotes disulfide bond formation similar to that in SHuffle T7 strain. Thus, this strategy of purification and refolding of SRCRD proteins from inclusion bodies expressed by SHuffle T7 strain is considered to be reliable.

**Table 4:** Characteristics of three constructs for SRCRD expression and crystallization

Constructs	Expression	Final yield	Crystallization^a^	Crystal type
Trx-His_6_-tev-SRCRD	Supernatant	Low	A few crystals	Needle-shaped crystal
His_6_-SRCRD	Inclusion body	High	No crystal	–
SRCRD	Inclusion body	High	Many crystals	Rhombic crystal

^a^For Trx-His_6_-tev-SRCRD construct, the Trx-His_6_ was removed by TEV protease digestion and SRCRD only was purified for crystallization. For His_6_-SRCRD construct, His_6_-SRCRD was used for crystallization. For SRCRD construct, SRCRD was used for crystallization

Regarding the crystal structure, by matching the electron density map, we confirmed the correct formation of all four pairs of disulfide bonds. SRCRD exhibits a highly conserved structure. In addition to the multiple cysteines that formed disulfide bonds, several amino acid sequences that constitute the primary secondary structure exhibited a very high level of conservation.

The disulfide bond formed by the C1–C4 pair distinguishes Type A from Type B SRCRDs [45]. This pair is located near the protein surface and may lead to the generation of inclusion bodies because of the faulty intermolecular bonds formed by it. By comparing the position of this extra disulfide bond pair and the nearby structure in the Type B SRCRD structure with those of Type A, we found that the C1–C4 pair may help link the terminal peptide chains and stabilize the structural closure together with the C3–C8 pair and nearby hydrogen bonds. These bonds link the N-terminal β-strands, C-terminal β-strand, and α-helix to each other. In the Type A structure, this function is performed independently by the C3–C8 pair and the hydrogen bonds between the β-strands. Overall, the C1–C4 pair causes almost no change in the SRCRD intact structure; rather, it only serves as an additional insurance for the closure of the structure, which also ensures the conservation between Type A and B domains. However, the extra two cysteines would likely be a factor in the production of inclusion bodies. In any case, this strategy of successfully expressing and refolding all disulfide bonds, including the C1–C4 pair, to obtain high-quality Type B SRCRD proteins exhibits high potential for widespread application to other both Type A and Type B SRCRD proteins.

In conclusion, our studies led to the development of a new and efficient strategy for the production of SRCRD proteins using an *E. coli* expression system. The recombinant protein containing four disulfide bonds could be refolded using a simple and efficient procedure, with the refolding buffer containing glutathione as a redox system. Crystallization and structural analysis confirmed the appropriate formation of disulfide bonds and the correct folding of the protein structure. The expression and purification strategy reported in this study may further contribute to the expression and functional study of other scavenger receptor family proteins and Cys-rich proteins.

### Supplementary Information

Below is the link to the electronic supplementary material.

Fig. S1 Identification of Trx-His6-TEV-SRCRD and purified SRCRD by MALDI-TOF-MS (TIF 165 KB)

Fig. S2 Size-exclusion chromatography of the RPC-purified SRCRD sample before and after refolding. Before refolding, most of the protein was observed to be eluted in the void volume as an aggregate. After refolding, most of the protein was observed to be eluted as a monomer (TIF 578 KB)

Fig. S3 Effect of DTT on the digestion of Trx-His6-TEV-SRCRD by TEV protease. Samples were prepared for SDS–PAGE using a non-reducing sample buffer. Different concentrations of DTT (0, 5, and 10 mM) were added to the protein samples before TEV protease digestion. The digested products were heat-treated and non-heat-treated, respectively (TIF 91 KB)

Fig. S4 Size-exclusion chromatography of refolded SRCRD. Most of the protein was observed to be eluted as a monomer (TIF 267 KB)

Fig. S5 SDS-PAGE of SRCRD expressed by E. coil BL21(DE3). “S” represents the soluble fraction of the cell lysate. “P” denotes the precipitate fraction of the cell lysate. “R” represents the sample solubilized in refolding buffer. “D” is the precipitate formed during dialysis after refolding (TIF 200 KB)

Fig. S6 The crystals of SRCRD obtained via the inclusion body expression strategy grew in several conditions of the PEG/ion kit. The conditions that favored SRCRD crystal growth are marked by green boxes (TIF 98 KB)
