# Supplementary figures and images for: Refolding, Crystallization, and Crystal Structure Analysis of a Scavenger Receptor Cysteine-Rich Domain of Human Salivary Agglutinin Expressed in Escherichia coli
Source: Protein J. 2024 Jan 24;43(2):283–97. doi: 10.1007/s10930-023-10173-x (PMC11058800; doi:10.1007/s10930-023-10173-x)

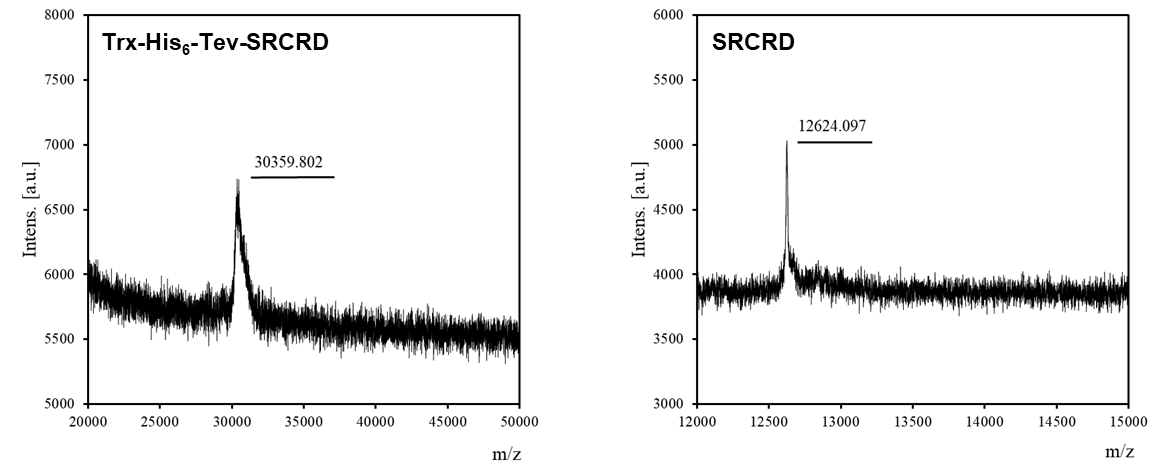

Supplement: Supplementary file 1 — Fig. S1 Identification of Trx-His6-TEV-SRCRD and purified SRCRD by MALDI-TOF-MS (TIF 165 KB) [file 10930_2023_10173_MOESM1_ESM.tif]

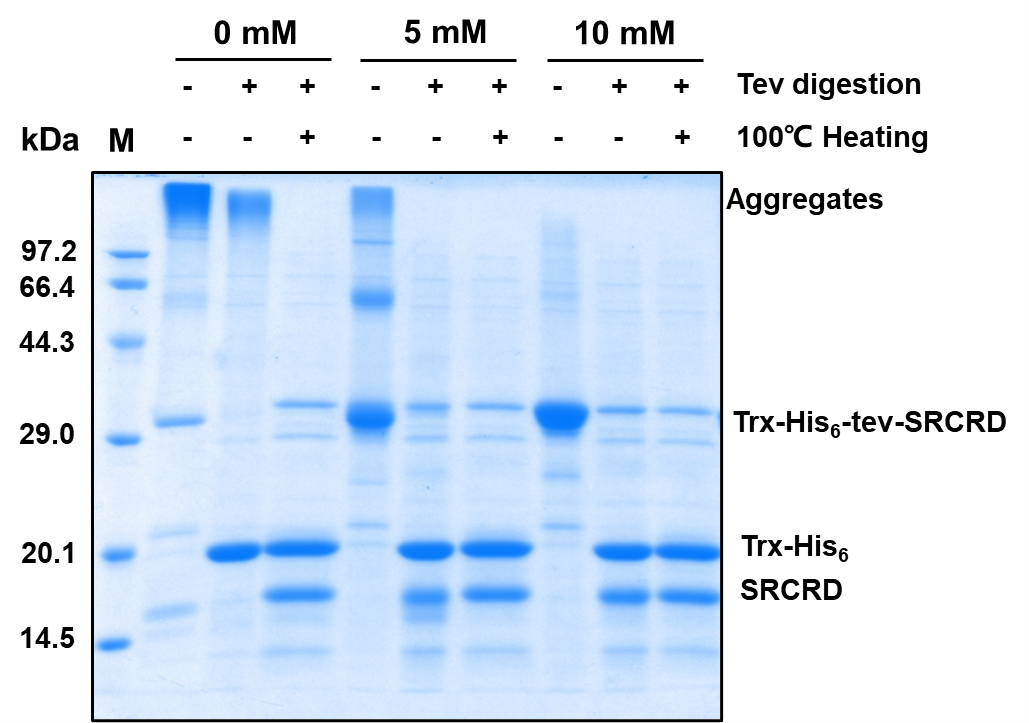

Supplement: Supplementary file 2 — Fig. S2 Size-exclusion chromatography of the RPC-purified SRCRD sample before and after refolding. Before refolding, most of the protein was observed to be eluted in the void volume as an aggregate. After refolding, most of the protein was observed to be eluted as a monomer (TIF 578 KB) [file 10930_2023_10173_MOESM2_ESM.tif]

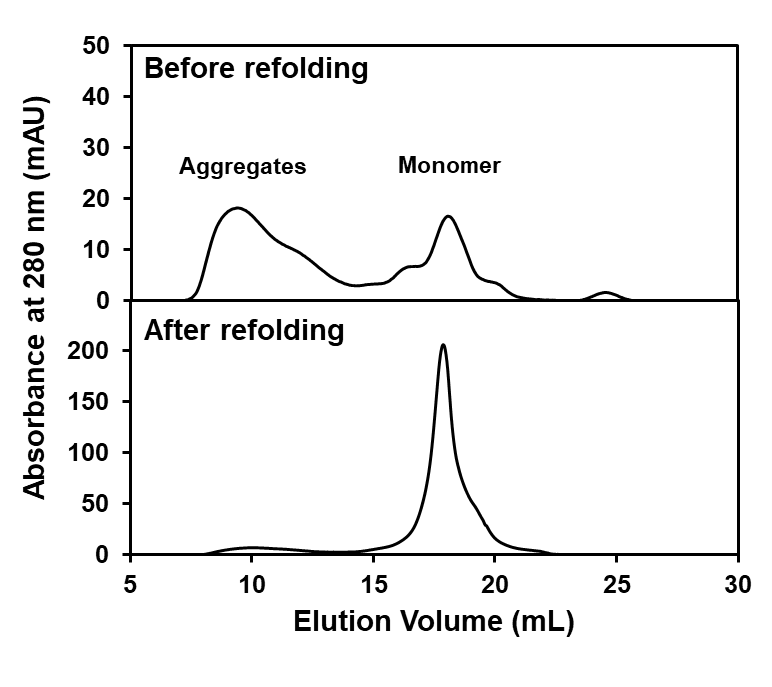

Supplement: Supplementary file 3 — Fig. S3 Effect of DTT on the digestion of Trx-His6-TEV-SRCRD by TEV protease. Samples were prepared for SDS–PAGE using a non-reducing sample buffer. Different concentrations of DTT (0, 5, and 10 mM) were added to the protein samples before TEV protease digestion. The digested products were heat-treated and non-heat-treated, respectively (TIF 91 KB) [file 10930_2023_10173_MOESM3_ESM.tif]

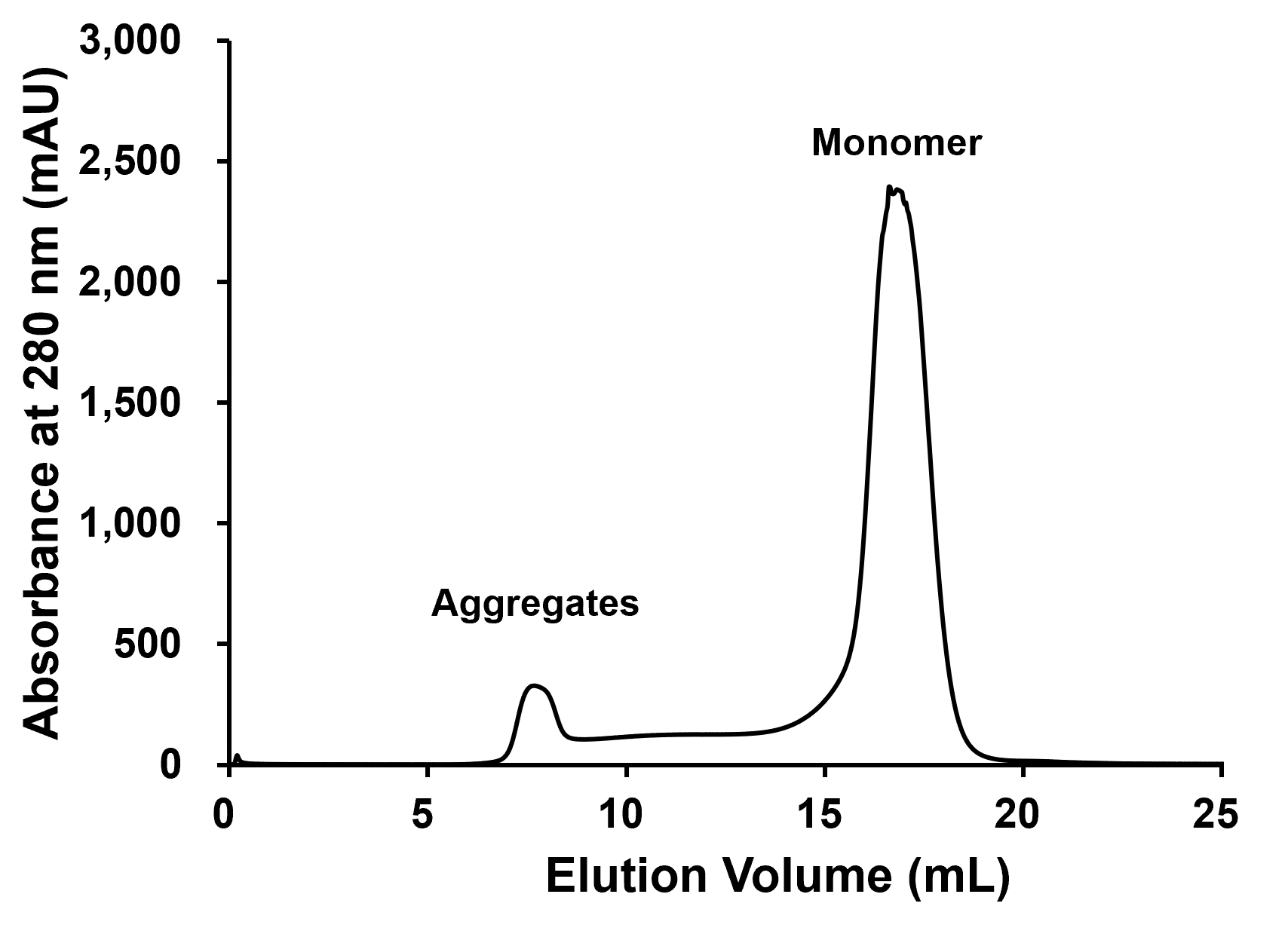

Supplement: Supplementary file 4 — Fig. S4 Size-exclusion chromatography of refolded SRCRD. Most of the protein was observed to be eluted as a monomer (TIF 267 KB) [file 10930_2023_10173_MOESM4_ESM.tif]

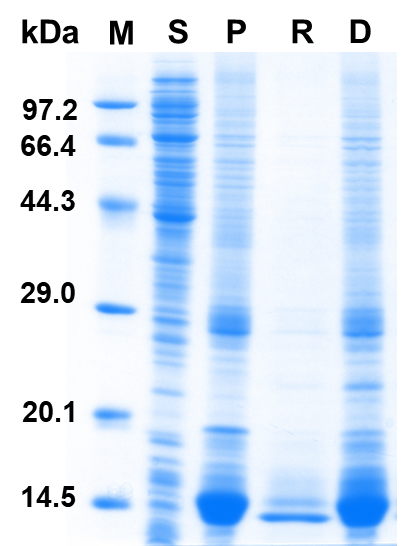

Supplement: Supplementary file 5 — Fig. S5 SDS-PAGE of SRCRD expressed by E. coil BL21(DE3). “S” represents the soluble fraction of the cell lysate. “P” denotes the precipitate fraction of the cell lysate. “R” represents the sample solubilized in refolding buffer. “D” is the precipitate formed during dialysis after refolding (TIF 200 KB) [file 10930_2023_10173_MOESM5_ESM.tif]

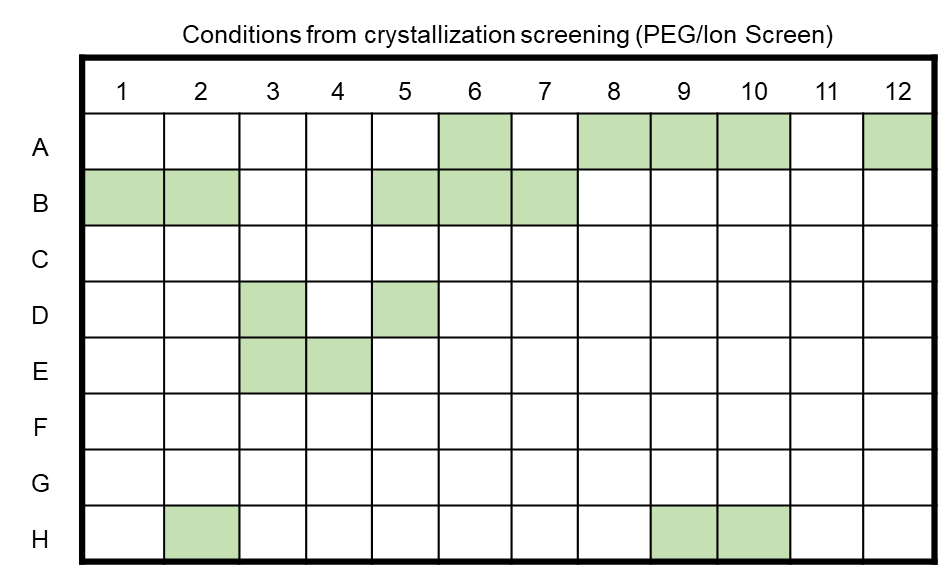

Supplement: Supplementary file 6 — Fig. S6 The crystals of SRCRD obtained via the inclusion body expression strategy grew in several conditions of the PEG/ion kit. The conditions that favored SRCRD crystal growth are marked by green boxes (TIF 98 KB) [file 10930_2023_10173_MOESM6_ESM.tif]
